# Phenotypic Variation in Mojave Rattlesnake (*Crotalus scutulatus*) Venom Is Driven by Four Toxin Families

**DOI:** 10.3390/toxins10040135

**Published:** 2018-03-23

**Authors:** Jason L. Strickland, Andrew J. Mason, Darin R. Rokyta, Christopher L. Parkinson

**Affiliations:** 1 Department of Biology, University of Central Florida, 4110 Libra Drive, Orlando, FL 32816, USA; Jason.Strickland@ucf.edu; 2 Department of Biological Sciences, Clemson University, 190 Collings St., Clemson, SC 29634, USA; ajmason@clemson.edu; 3 Department of Biological Science, Florida State University, 319 Stadium Drive, Tallahassee, FL 32306, USA; drokyta@bio.fsu.edu

**Keywords:** C-type lectins, hemorrhagic, Mojave toxin, myotoxin *a*, neurotoxic, phospholipase A_2_, RNA-seq, snake venom metalloproteinases

## Abstract

Phenotypic diversity generated through altered gene expression is a primary mechanism facilitating evolutionary response in natural systems. By linking the phenotype to genotype through transcriptomics, it is possible to determine what changes are occurring at the molecular level. High phenotypic diversity has been documented in rattlesnake venom, which is under strong selection due to its role in prey acquisition and defense. Rattlesnake venom can be characterized by the presence (Type A) or absence (Type B) of a type of neurotoxic phospholipase A${}_{2}$ (PLA${}_{2}$), such as Mojave toxin, that increases venom toxicity. Mojave rattlesnakes (*Crotalus scutulatus*), represent this diversity as both venom types are found within this species and within a single panmictic population in the Sonoran Desert. We used comparative venom gland transcriptomics of nine specimens of *C. scutulatus* from this region to test whether expression differences explain diversity within and between venom types. Type A individuals expressed significantly fewer toxins than Type B individuals owing to the diversity of C-type lectins (CTLs) and snake venom metalloproteinases (SVMPs) found in Type B animals. As expected, both subunits of Mojave toxin were exclusively found in Type A individuals but we found high diversity in four additional PLA${}_{2}$s that was not associated with a venom type. Myotoxin *a* expression and toxin number variation was not associated with venom type, and myotoxin *a* had the highest range of expression of any toxin class. Our study represents the most comprehensive transcriptomic profile of the venom type dichotomy in rattlesnakes and *C. scutulatus*. Even intra-specifically, Mojave rattlesnakes showcase the diversity of snake venoms and illustrate that variation within venom types blurs the distinction of the venom dichotomy.

## 1. Introduction

Differential gene expression is a primary component with respect to the genotype and phenotype that facilitates rapid evolutionary response in the face of changing environmental pressures by generating phenotypic diversity [1]. Comparative transcriptomics has emerged as the tool to understand these responses by linking the phenotype to the genotype through mRNA sequencing. However, the molecular mechanisms underlying phenotypic divergence are difficult to determine because there is usually no one-to-one link between the genotype and phenotype due to pleiotropic and epistatic effects [2,3]. Venom is an exception to this because it is a complex trait that is highly tractable from the gene being expressed to the final protein product [4]. Venom is under strong selection as it aids in prey acquisition and/or serves as a predator deterrent [5]. Changes within the venom phenotype occur through regulatory shifts in protein expression [6], through loss of specific genes [7], duplication [8], and point mutations [9]. Transcriptomics cannot detect all possible mechanisms resulting in phenotypic diversity, particularly with regard to genes in the genome that are not expressed or have multiple copies [10]. However, for those genes that are expressed, transcriptomics offer an effective means of linking sequence-based and regulatory variation to changes in a composite phenotype [11]. With the increase in availability of high-throughput proteomic, transcriptomic, and genomic resources, rattlesnakes and their venom have become a model system to understand these mechanisms as they exhibit high phenotypic diversity [12,13,14,15].

Rattlesnake venoms can be broadly characterized by the presence or absence of heterodimeric phospholipases A${}_{2}$ (PLA${}_{2}$s). Type A venoms contain this PLA${}_{2}$ which is a β-neurotoxin responsible for highly toxic venom in individuals that express it. These venoms also have little hemorrhagic activity due to low expression of snake venom metalloproteinases (SVMPs). Type B venoms lack the neurotoxic PLA${}_{2}$ and are also characterized by high hemorrhagic activity due to high expression of SVMPs [12,13,15,16,17]. When present, the PLA${}_{2}$ acts presynaptically to disrupt the nervous system and both the acidic and basic subunits of the heterodimer must be expressed in the venom for the neurotoxic effect to occur. The origin of this toxin and its effect is due to a single nucleotide substitution that allowed the interaction of the two subunits to be energetically favorable [9], and no evidence exists of this PLA${}_{2}$ being in the genome and not being expressed proteomically [18,19,20]. Examples include Mojave toxin (MTX) in *Crotalus scutulatus* and its close relatives, sistruxin in *Sistrurus catenatus*, crotoxin in the *Crotalus durissus* complex, and canebrake toxin in *Crotalus horridus* [21,22,23,24,25]. Of the 48 species of rattlesnakes (*Crotalus* and *Sistrurus*) currently recognized [26], 38 are considered as Type B, one is Type A, and 9 are documented as polymorphic [25].

The lack of a phylogenetic pattern in venom phenotypes exhibited by rattlesnakes has hampered our understanding of the evolution of this dichotomy. The most recent hypothesis, based on genomic sequencing and ancestral state reconstruction, is that the ancestral state was neurotoxic (Type A) and lineages no longer possessing the neurotoxic PLA${}_{2}$ (hereafter Mojave toxin/MTX) have lost it [7]. Dowell et al. [7] examined three rattlesnake species (*Crotalus adamanteus*, *Crotalus atrox*, and *C. scutulatus*) and found that these three species have different sets of PLA${}_{2}$s based on venom type, which is supported by current transcriptomic evidence. Rokyta et al. [27] compared the venom gland transcriptomes of one individual from a Type B species (*C. adamanteus*) to one Type A species (*C. horridus*) and found different sets of PLA${}_{2}$s. This was further supported by Rokyta et al. [28] when they compared the venom gland transcriptomes of one Type B individual and one Type A individual of *C. horridus* to examine intraspecific variation. They found different sets of PLA${}_{2}$s expressed in the transcriptome between Type A and Type B *C. horridus*. Additionally, both studies found evidence to support the dichotomy. Type A individuals had simpler venom in that fewer toxins were expressed and the primary difference was the trade-off between MTX and SVMPs, but they also found differences in C-type lectins (CTLs) and myotoxin *a* (hereafter MYO or myotoxins) [27,28]. Like *C. horridus*, *C. scutulatus* (Dowell et al. [7]’s neurotoxic/Type A representative), have well documented intraspecific venom variability corresponding to the Type A and Type B venom phenotypes throughout their distribution [20,21,29,30,31,32,33].

**Figure 1:**
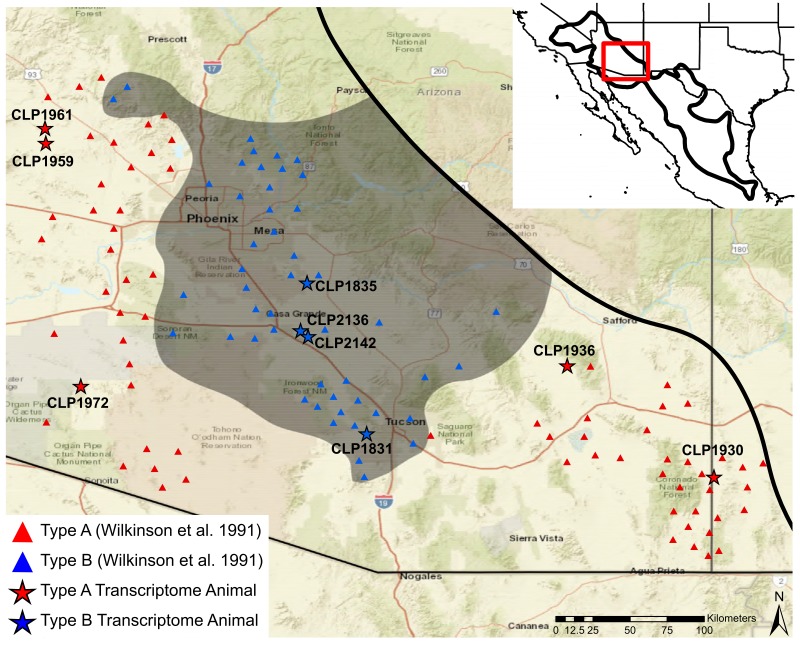
Distribution of Type A and Type B venom in Arizona based on data from Wilkinson et al. [31] (triangles) and the nine transcriptome animals sequenced (stars). The shaded area is the estimated distribution of Type B venom and the dark black line is the outline of the estimated distribution of *Crotalus scutulatus*. CLP: Christopher L. Parkinson field number.

Mojave rattlesnakes (*C. scutulatus*) are known to be present from the deserts of the southwestern United States and as far south as the state of Puebla in Mexico (Figure 1). *Crotalus scutulatus* is comprised of three phylogeographic lineages [34]. The basal lineage includes the subspecies *Crotalus scutulatus salvini*, which has Type A venom and is located at the southern end of the distribution [12,33]. The remaining two lineages are *Crotalus scutulatus scutulatus* distributed in the Chihuahuan Desert and Sonoran/Mojave Deserts (hereafter, Sonoran), respectively [34]. Both Type A and Type B venoms are found in the latter two lineages, and although rare, snakes possessing a Type A + B phenotype (highly expressing both MTX and SVMPs) have also been documented [18,20,31,35,36].

The venom phenotype complexity in *C. scutulatus* is best documented within the Sonoran lineage [37,38,39]. Proteomic differences in the venom seem to be geographically fixed, but intergradation occurs where Type A and Type B ranges come into contact [12,31,35,39,40]. In addition to the dichotomy between PLA${}_{2}$s and SVMPs, variability in other toxins has been documented [36,39]. Particularly, Massey et al. [39] described variation in myotoxins in one small area of the distribution where some individuals had ∼25% of their proteome made up of myotoxins. This finding prompted them to suggest further dividing venom types within *C. scutulatus* into Types A–F to account for variation between PLA${}_{2}$s, SVMPs, and myotoxins, and hypothesize that myotoxins occur when the Type A and Type B phenotypes come into contact [39]. No phylogeographic structure exists and high gene flow occurs between individuals regardless of venom type within this region based on allozymes [31], mitochondrial (ND4), and nuclear (RADSeq) data [34], so genetic recombination at the contact zone is possible.

To examine the role of differential expression in the evolution of venom phenotypes, we used Sonoran Desert Mojave rattlesnakes because they are a microcosm for the diversity found in rattlesnakes. Within the Sonoran lineage, high venom diversity exists and six phenotypes are described [36,39] with Type A and Type B venom phenotypes being geographically fixed despite high gene flow [31,34]. Moreover, the ancestral lineage within Mojave rattlesnakes, *C. scutulatus salvini*, is neurotoxic [33,34], as is the hypothesized ancestral rattlesnake [7]. Thus, mechanisms underlying the presence or absence of Mojave toxin in other rattlesnake species may become more apparent by focusing on *C. scutulatus*. Through comparative venom-gland transcriptomics, we link the patterns found genomically and the diversity identified proteomically to test: (1) whether myotoxin expression is localized to contact areas between individuals with Type A and Type B venom; (2) if expression patterns within and between venom phenotypes are consistent among species and individuals; and (3) whether individuals with Type A and Type B venom will express distinct sets of PLA${}_{2}$s as hypothesized by Dowell et al. [7]. In this pursuit, we present the most extensive transcriptomic sampling to date of *Crotalus scutulatus* and the A/B venom dichotomy.

## 2. Results

### 2.1. Venom Gland Transcriptomes of *C. scutulatus*

**Figure 2:**
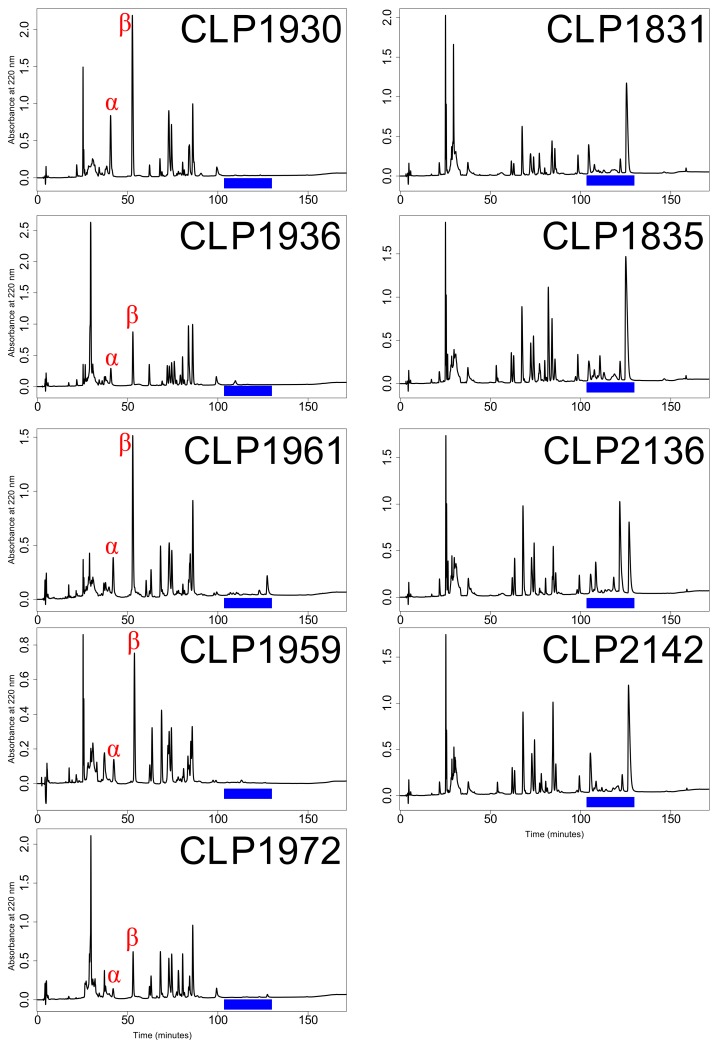
RP-HPLC profiles for the nine specimens of *C. scutulatus* selected for transcriptome sequencing. Type A individuals are in the left column and Type B individuals are in the right column. α = acidic subunit of Mojave toxin; β = basic subunit of Mojave toxin; Blue bar = region where SVMPs elute.

We sequenced the venom gland transcriptomes of nine *C. scutulatus* individuals from the Sonoran Desert in the U.S.A. (Table 1, Figure 1). These individuals were chosen after determining their venom phenotype using RP-HPLC (Figure 2) to maximize the venom variation in *C. scutulatus*. Using 150 bp paired-end transcriptome sequencing on the Illumina MiSeq and HiSeq platforms, we generated over 131 million raw read pairs that yielded over 113 million merged reads that passed the quality filter, and where the 3${}^{\prime }$ ends overlapped (Table 1). The nine individuals had an average of 12.6 ± 3.7 million merged reads. After assembly, annotation, duplicate and chimera removal, and clustering, our consensus *C. scutulatus* transcriptome consisted of 1889 putative nontoxins and 75 putative toxins from 17 toxin families (Table 2). To be considered present in the transcriptome, toxins had to have at least 5× read coverage over 90% of the total transcript sequence after mapping. Of the 75 toxins identified, three were exclusively found in animals with Type A venom and 17 were exclusively found in individuals with Type B venom (Table 3). Type A individuals had an average of 48.6 ± 6.1 toxins and Type B individuals had 65.6 ± 2.4 toxins. This difference was found to be significant based on a Mann–Whitney–Wilcoxon test (W = 0, df = 7, *p* = 0.019). Only 33 toxins were in all individuals and another 22 toxins were found in both Type A and Type B individuals but not in all nine individuals (Table 3).

### 2.2. Toxin Diversity in *C. scutulatus*

**Figure 3:**
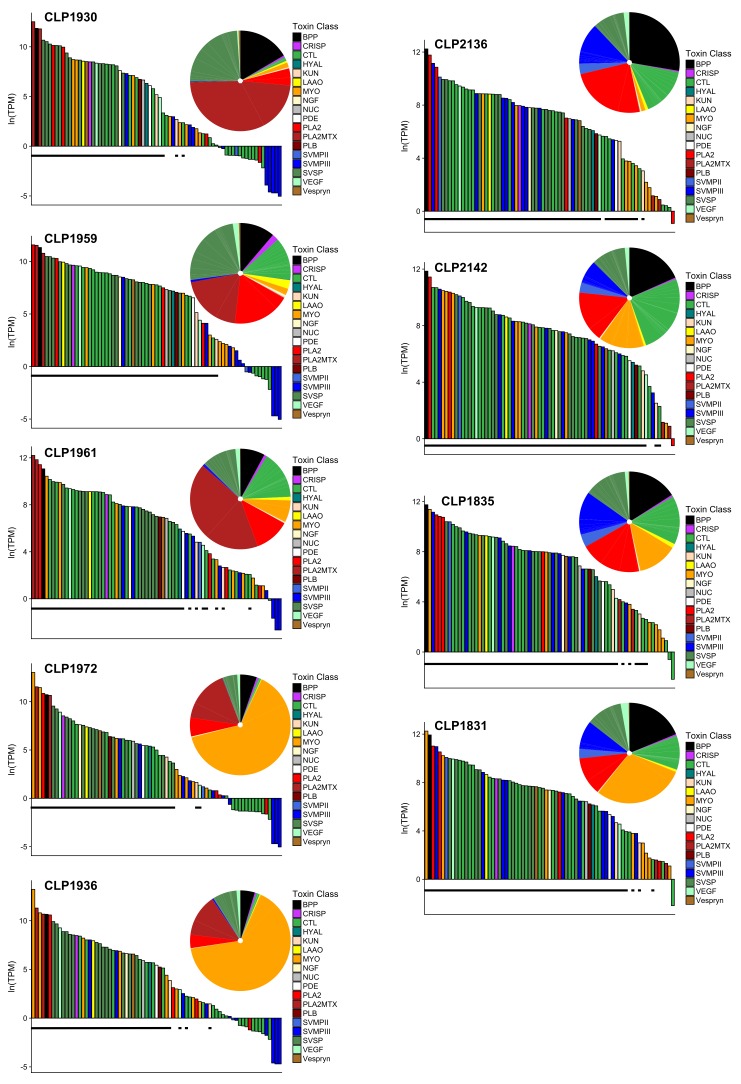
Representation of the toxins in the venom gland transcriptome for nine *C. scutulatus* specimens. Type A are on the left and type B are on the right, and are ranked by the increasing amount of myotoxins. The bar graphs represent each of the 75 toxins identified. Any toxin that does not have a black bar under it did not meet the criteria for presence in the transcriptome. The pie charts represent the proportion of each toxin family in the venom gland transcriptome.

**Figure 4:**
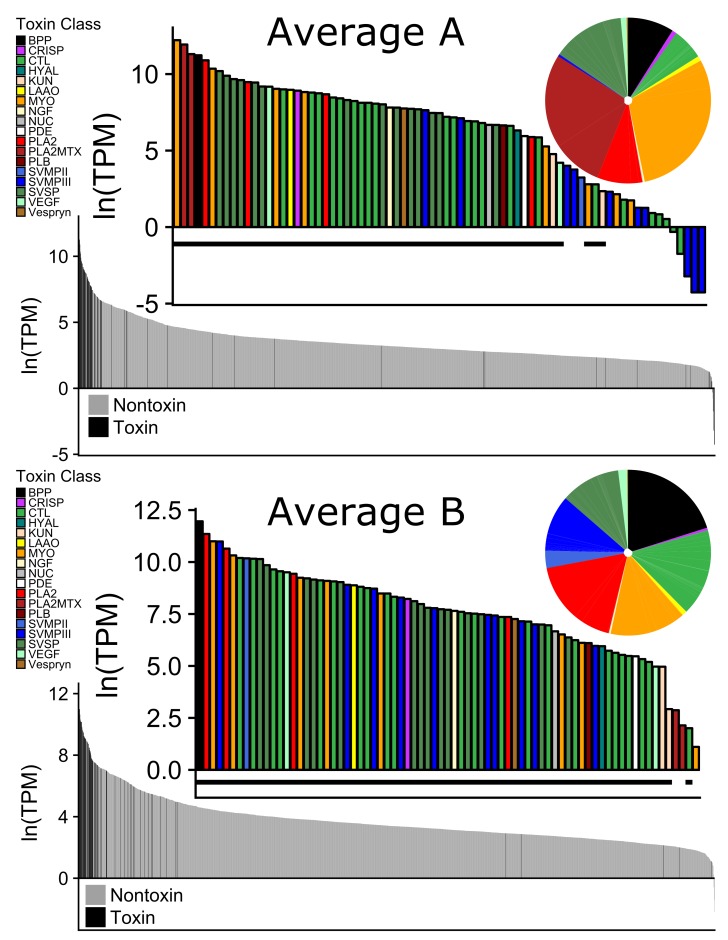
Representation of the average Type A and Type B venom gland transcriptome of *C. scutulatus* from the Sonoran Desert. These were generated by averaging the TPM values of each individual within a venom type. For both Type A and Type B, the majority of the highly expressed transcripts are toxins. The bar graphs with colors represent each of the 75 toxins identified. Any toxin that does not have the black bar under it did not meet the criteria for being present in the transcriptome of any individual of that venom type. The pie charts represent the proportion of each toxin family in the venom gland transcriptome. Type A individuals have very few SVMPs and the Type B individuals are lacking neurotoxic phospholipase A${}_{2}$ (PLA${}_{2}$), the Mojave toxin.

We found high intraspecific diversity in the venom gland transcriptome both within and between Type A and Type B venoms in *C. scutulatus* (Figure 3). Much of the diversity was due to the presence and expression differences between Type A and Type B individuals in three toxin families: PLA${}_{2}$s (including MTX), SVMPs, and CTLs. However, we document the first case of CTLs being highly expressed in the transcriptome of Type A animals. When comparing the average transcriptomes for Type A and Type B, the dichotomy in toxins is clear (Figure 4). However, there were toxins that demonstrated high variability in presence and expression both within and among venom types. Myotoxins had the most variation in expression of a toxin family not associated with venom type (Figure 3). In two Type A individuals (Christopher L. Parkinson field number CLP1972 and CLP1936) and one Type B individual (CLP1831), MYO-1 was the most highly expressed toxin in the transcriptome and was the second highest in CLP1835 (Figure 3). The most diverse toxin family was the CTL family, with 23 different putative toxins identified, followed by snake venom serine proteases (SVSPs) with 14, SVMPIIIs with 10, and myotoxins with 8 (Table 2).

### 2.3. Myotoxin *a* Diversity

Myotoxins were the fourth most diverse toxin family with eight different toxins identified and presence and expression levels were highly variable among individuals (Figure 3). The four most highly expressed myotoxins were not associated with venom type nor were myotoxins associated with contact zones between the two types. CLP1835 had 10.1% of its toxin expression comprised of myotoxins and was closest to the center of the Type B distribution. Additionally, CLP2136 and CLP2142 were found very close to each other but CLP2136 had little if any myotoxin expression. The percentage of myotoxins in the toxin transcriptome ranged from 1.0% to 60.4% and was not associated with size or sex.

### 2.4. Expression Differences in Type A and Type B C. scutulatus

**Figure 5:**
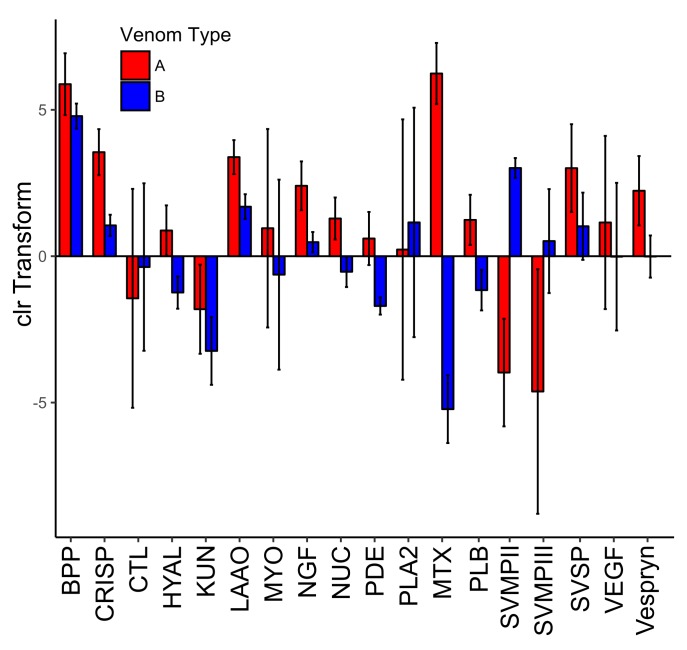
Average expression of toxin families with respect to Type A and Type B *C. scutulatus* after centered log-ratio (clr) transform. Error bars are the standard deviation around the mean of the clr-transformed data.

Toxin family expression levels were highly variable among families and many differed between venom types. (Figure 5). The PLA${}_{2}$s that comprise MTX were highly expressed in Type A, with very low expression in Type B as expected. The opposite was true for the SVMPII and the SVMPIIIs. These correspond to the major differences between Type A and Type B venom (Figure 4 and Figure 5). For most of the toxin families there was variability within venom types as illustrated by the error bars in Figure 5. The non-MTX PLA${}_{2}$s, CTLs, MYOs, and vascular endothelial growth factors (VEGFs) all had almost completely overlapping standard deviations. Other toxin families such as bradykinin potentiating peptide (BPP), cysteine-rich secretory protein (CRISP), l-amino-acid oxidase (LAAO), and nerve growth factor (NGF) did differ between venom types slightly but had much tighter variation in expression levels within each venom type.

Using the pairwise comparisons of all Type A individuals to all Type B individuals (20 comparisons), we found many toxins that differed between Type A and Type B individuals (Table 4). Many of these corresponded with the presence/absence variation between PLA${}_{2}$s and SVMPs but others were in toxins expressed in all individuals. Our DESeq1 and DESeq2 analyses were more conservative in identifying toxins that differed between Type A and Type B venoms, primarily identifying toxins that had dramatic presence/absence differences in the transcriptome. DESeq1 identified 20 toxins and DESeq2 identified 26 toxins that were significantly different but 13 and 19 were toxins that were exclusively found in one venom type by each program, respectively. Only Pla2gB1, SVSP-2, and CTL-12 were identified among the remaining toxins by both analyses. Of the 33 toxins present in all nine individuals, only CRISP-1, phospholipase B (PLB-1), and SVSP-5 were identified as differentially expressed in DESeq2. By comparison, the only toxin identified as having significantly different expression between males and females in DESeq1 and DEseq2 was PLA2gA1, which is likely an artifact of sampling rather than an indication of sexual dimorphism.

**Figure 6:**
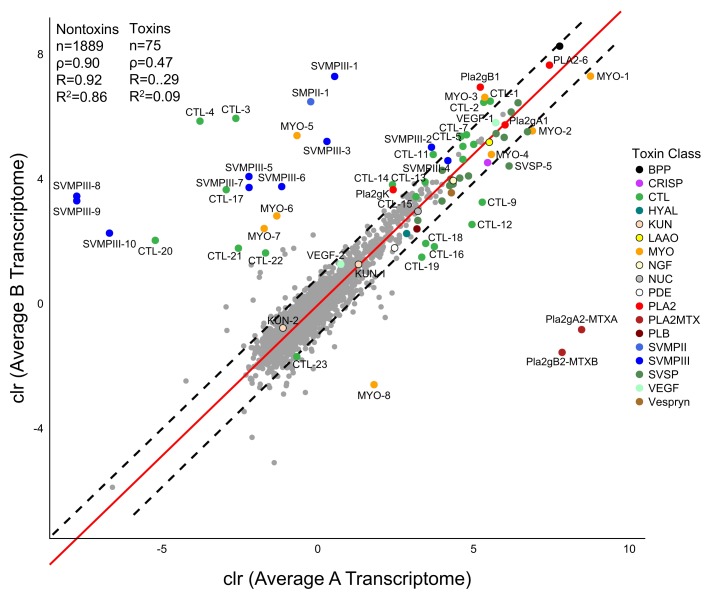
Pairwise comparison of the average Type A and Type B venom gland transcriptomes using the centered log-ratio (clr)-transformed TPM data. SVSPs and toxin families that only have one toxin are not labeled. SVSPs did not differ between the two transcriptomes. The red line is the line of best fit through the non toxins and the dashed black lines are the 99% confidence around that line. Any transcript outside the dashed black lines was identified as an outlier. Anything above the upper line is overexpressed in Type B and anything below the lower line is overexpressed in Type A.

For the pairwise comparison of the Average A and Average B transcriptomes, the relative expression of each of the nontoxins was highly correlated (Figure 6). However, toxin expression between venom types was poorly correlated. This was driven by the two subunits of MTX, SVMPs, CTLs, and myotoxins. The remaining toxins within the toxin families were highly correlated between the venom types. This includes BPP, CRISP, hyaluronidase (HYAL), Kunitz peptide (KUN), LAAO, NGF, 5${}^{\prime }$ nucleotidase (NUC), phosphodiesterase (PDE), PLB, SVSPs, VEGF, and Vespryn.

### 2.5. PLA${}_{2}$ Diversity

Dowell et al. [7] annotated nine PLA${}_{2}$s with mRNA coding sequence in the four sequenced genome fragments. Of these, only five were expressed in the venom gland transcriptome and the remaining four were exclusively found in the genomes of the species they sequenced (see Figure 1 in [7]). We found evidence for all five of the expressed PLA${}_{2}$s in our transcriptomes as well as a sixth PLA${}_{2}$ (PLA${}_{2}$-6) not recovered in Dowell et al. [7] (Table 5). As expected, the two ancestral mammal homolog PLA${}_{2}$s, Pla2-e and Pla2-f, were not expressed in the venom gland. In agreement with Dowell et al. [7] we did not find evidence for Pla2-gC1 identified in all three genomes nor Pla2-d identified in the *C. adamanteus* genome being expressed. No PLA${}_{2}$ was found in all individuals and one individual (CLP1959) had evidence for all six PLA${}_{2}$s being present although Pla2gA1 had very low expression compared to other individuals and PLA${}_{2}$s (Table 5). In CLP1835, the sequence for the Pla2gA1 was not the same as the other *C. scutulatus* individuals that expressed it. When blasted against the non-redundant nucleotide database in GenBank, it matched the sequence of *Crotalus viridis* (Accession AF403134) which is in the sister species complex to *C. scutulatus*. The sequence had eight nonsynonymous nucleotide changes compared to the other three *C. scutulatus* individuals that expressed Pla2gA1 (Table 5).

## 3. Discussion

The major differences in the venom gland transcriptome within and between Type A and Type B *C. scutulatus* from the Sonoran Desert were driven by the presence or absence of PLA${}_{2}$s including Mojave toxin (MTX), snake venom metalloproteinases (SVMPs), C-type lectins (CTLs), and myotoxins (MYO) (Figure 3). Myotoxin expression was not associated with the contact zones between the two venom types as hypothesized by Massey et al. [39]. We did not find evidence for distinct sets of PLA${}_{2}$s for Type A and Type B venom types as hypothesized by Dowell et al. [7] using three species (two Type B/one Type A). Additionally, one individual expressed all six PLA${}_{2}$s (Table 5). We also found evidence for a PLA${}_{2}$ allele in one *C. scutulatus* individual that was more similar to a congener, *C. viridis*, than the other eight *C. scutulatus* specimens. The allele could have originated from interspecific hybridization with a member of the *C. viridis* species complex. Other works have suggested introgression through hybridization as a mechanism for propagating toxin genes among species. For example, It is hypothesized that the *C. horridus* MTX homolog, canebrake toxin, was introduced by intergeneric hybridization with *Sistrurus catenatus* [28]. These data, taken together, support non-allelic homologous recombination (NAHR) as an important mechanism driving PLA${}_{2}$ diversity in *C. scutulatus* venom and rattlesnakes more broadly [7].

Expression differences between Type A and Type B venom were due to the presence/absence of specific toxins (Table 4). Type A venoms were simpler in that they contained fewer toxins overall, driven by the lack of SVMP and CTL expression. The presence/absence expression difference between MTX and SVMPs is the characteristic difference between the Type A (neurotoxic) and Type B (hemorrhagic) venom dichotomy seen within rattlesnakes. As expected, both subunits of the neurotoxic PLA${}_{2}$ (Mojave toxin) were exclusively found in Type A individuals, and Type B individuals had a high diversity of SVMPs [43,44]. All but two SVMPs (SVMPIII-2 and SVMPIII-4) were absent from all Type A individuals which is similar to what was found in the Type A *C. horridus* [28]. SVMPIII-4 was found in all nine individuals and SVMPIII-2 was missing in CLP1930 (Table 3).

C-type lectins (CTLs) had the highest number of unique toxins within a family, with 23 different toxins (Table 2). Only two were found in all nine individuals, six were exclusively found in Type B individuals, and the remaining 15 were found in different combinations regardless of venom type (Table 3). CTLs are diverse in Type B species and affect coagulation factors, increasing hemorrhaging [27,45]. Interestingly, two of the Type A individuals, CLP1959 and CLP1961, had high expression of CTLs at 10.2% and 12.4%, respectively where the other three individuals had almost no expression (≤1.0%, Figure 3). This is the first time that CTLs have been documented to be highly expressed in a Type A individual. In the Type A *C. horridus*, CTLs only accounted for 0.2% of the toxin reads [27].

Massey et al. [39] documented high intraspecific variability of myotoxin in the proteome of *C. scutulatus*, independent of venom type. Because of this, they suggested *C. scutulatus* venoms be further divided into six venom types: Type A, Type A + M, Type B, Type B + M, Type A + B, and Type A + B + M. The addition of these myotoxins in the venom decreased the lethal dose 50 (LD${}_{50}$) values and work to disrupt sodium channels in muscle cells causing muscle paralysis [39,46]. Our transcriptome data support further differentiating venom types to account for the diversity in myotoxin expression levels. We found differences in myotoxin expression among the nine individuals but they were not associated with the contact zone between the two venom types as predicted. Of the eight myotoxins we identified, one was found exclusively in Type A individuals and two were exclusively found in type B individuals (Table 3). However, these are likely a function of sampling rather than fixed toxins in those venom types given the variability in myotoxins overall (Figure 3). When present, the four most highly expressed myotoxins (MYO-1–4) were expressed at similar levels between the venom types (Figure 6).

Based on the hypothesis of Dowell et al. [7], we expected to find distinct sets of PLA${}_{2}$s in Type A and Type B *C. scutulatus*, as was found in *C. horridus* [28] and the three species with genomic fragments sequenced [7]. However, this was not the case and we found almost all possible combinations of the six putative PLA${}_{2}$ toxins among the nine individuals we sequenced (Table 5). Other than the acidic and basic subunits of MTX which were exclusively found in Type A individuals, the remaining four PLA${}_{2}$s were not specific to a venom type (Table 5). Further genomic analysis is needed to determine why the two subunits of MTX appear to be consistently found together whereas the other four can be inherited in different combinations. Additionally, it is possible for homologs of MTX, like crotoxin, to be present in the genome and not be expressed as *Crotalus simus* undergoes an ontogenetic shift where crotoxin is expressed in juveniles, but not expressed in adults [47].

One individual, CLP1959, expressed all six PLA${}_{2}$s including the acidic subunit of Mojave toxin (MTXA) and the basic unit of Mojave toxin (MTXB) and the PLA${}_{2}$s associated with Type B individuals, although one, Pla2gA1, was expressed at a significantly lower level than the other individuals that expressed it. That same toxin, Pla2gA1, in CLP1835 was 100% identical to a known allele found in *C. viridis*. All eight nucleotide changes from the other *C. scutulatus* that expressed it were non-synonymous, thus changing the amino acid sequence. The *C. viridis* species complex is sister to *C. scutulatus* so this could be a shared ancestral allele or introduced through hybridization. Hybridization between *C. scutulatus* and *C. viridis* has been documented but they are not syntopic in this region [19]. However, *Crotalus cerberus*, a member of the *C. viridis* species complex, is co-distributed with *C. scutulatus* in this region and could be the origin of this allele if it is shared within the complex.

Using two Type B species (*Crotalus atrox* and *C. adamanteus*) and one Type A individual (*C. scutulatus* from the Chihuahuan phylogeographic lineage), Dowell et al. [7] predicted that the region in the genome that contains the PLA${}_{2}$ genes is prone to NAHR and hypothesized that there would likely be diversity within a species as we document in *C. scutulatus*. If NAHR is the mechanism for gene movement, then it might explain how the different PLA${}_{2}$ genes, particularly MTX, can be reintroduced into populations that lose it through hybridization as hypothesized by Rokyta et al. [28] for *C. horridus*. *C. scutulatus* is known to hybridize with other species of rattlesnakes and, when this occurs, MTX can be found in the resulting hybrids [19]. Dowell et al. [7] agree with Lynch [48] in that PLA${}_{2}$s could go through a selective sieve after an ecological shift such as changing diet which causes the loss of the less adaptive PLA${}_{2}$s. This mechanism presumes SVMPs are down regulated or lost when MTX is present, but it could be the opposite. Alternatively, the two phenotypes may represent two fitness optima that can be maintained spatially or certain venom components could be selected for or against in specific environments based on different prey availability. Regardless of the mechanism, the interplay between gene flow and selection in the Sonoran Desert is allowing individuals with the two venom types to persist spatially without an obvious ecological difference between venom phenotypes.

The 13 remaining toxin families did not show the same pattern of presence/absence and all 27 toxins were present in each transcriptome at varying levels (Table 3 and Figure 4). Though snake venom serine proteinases (SVSPs) were the second most diverse toxin family, there were no differences in expression of the toxins between venom types. This is similar to what was found in *C. horridus* (see Figure 4 in Rokyta et al. [28]). There was one BPP and it was among the most highly expressed proteins in the venom of all individuals and had low variability among individuals (Figure 5). Other than KUN and VEGF, the remaining families (CRISP, LAAO, NGF, NUC, PDE, PLB, and Vespryn) also had low variability among individuals (Figure 5). We did not find any expression differences associated with the size or sex of *C. scutulatus*. Nontoxin expression was strongly correlated between the two venom types (Figure 4).

Mojave rattlesnakes are representative of the diversity documented in other rattlesnakes and our work illustrates the utility of sequencing multiple individuals of a species to represent the phenotypic diversity found. Both CTL and MYO diversity would have been underestimated within venom types if multiple individuals were not included (Figure 3). Overall, the transcriptomic differences in *C. scutulatus* matched the patterns documented between *C. adamanteus* and *C. horridus* [27] and that between Type A and Type B *C. horridus*. This included some individuals that had high levels of myotoxins as in *C. adamanteus* [49]. However, *C. scutulatus* was different in that myotoxin expression was much higher and there were no distinct sets of PLA${}_{2}$s between venom types as exhibited by the polymorphic *C. horridus*. *Crotalus scutulatus* will continue to be an exemplary model system to understand the evolution of venom particularly when the remaining phylogeographic lineages are included as well as the Type A + B individuals. Additionally, given the diversity in presence/absence of toxins within the major families, *C. scutulatus* would be useful to test NAHR in other toxin families.

## 4. Conclusions

Phenotypic diversity in *Crotalus scutulatus* is representative of venom diversity in rattlesnakes with both sequence-based and expression evolution occurring. By sampling and sequencing the transcriptome of multiple individuals of each venom phenotype, we were able to highlight the diversity throughout the Sonoran lineage, including the characteristic A and B venom phenotypes. For toxins that were not exclusively associated with venom type, different combinations occurred, particularly in PLA${}_{2}$s. High gene flow in this region of *C. scutulatus*’ distribution and interspecific hybridization may facilitate different combinations of these toxins, especially if NAHR occurs broadly among toxin families. Further genomic resources coupled with transcriptomics and proteomics of the remaining two lineages as well as the A + B phenotype may make it possible to understand the evolution of toxin gains and losses in PLA${}_{2}$s, CTLs, myotoxins, and SVMPs as well as differential expression and sequence evolutions of specific toxins.

In *C. scutulatus*, PLA${}_{2}$s, SVMPs, CTLs, and MYOs are primarily responsible for the transcriptomic diversity within and between the neurotoxic (Type A) and hemorrhagic (Type B) venom phenotypes. Many, but not all toxins within these four families are associated with the difference between the two phenotypes. Variation in myotoxins was found across the sampled range of *C. scutulatus* irrespective of venom type and was not exclusively found at contact zones between the two types. Their diversity among the nine individuals supports the further division of venom types within *C. scutulatus* and potentially all rattlesnakes to account for myotoxin diversity and expression level which is obscured when only considering the relationship between MTX and SVMPs. Our work represents the first complete venom gland transcriptome analysis in *C. scutulatus* and the best representation of species polymorphic for both Type A and Type B venoms. These data support utilizing Mojave rattlesnakes as a model for understanding the molecular mechanisms driving the evolution of phenotypic diversity.

## 5. Materials and Methods

### 5.1. Ethics Statement

Scientific collecting permits were issued by the New Mexico Department of Game and Fish (3563, 3576) and the State of Arizona Game and Fish Department (SP628489, SP673390, SP673626, SP715023). All interactions with animals were approved by the University of Central Florida’s Institutional Animal Care and Use Committee under protocol 13–17 W and followed the American Society of Ichthyologists and Herpetologists ethical guidelines.

### 5.2. Sample Collection

In the summers of 2013–2015, we collected *Crotalus scutulatus* from the Sonoran Desert in Arizona and New Mexico. In sampling, we targeted areas described by Wilkinson et al. [31] to have individuals with either Type A or Type B venoms (Figure 1). We collected a total of 42 individuals and processed them in preparation for venom gland transcriptome sequencing. First, we collected venom from each animal by tubing the individual in polycarbonate tubes (Get Hooked L.L.C., Sanford, FL, USA) and allowed them to move through the tube until just their head was protruding. Then, they were able to voluntarily bite a sterile collection cup covered in parafilm. Venom was collected, vacuum dried, and stored at −80 ${}^{\circ }$C for future use. Four days after venom was collected and transcription was maximized [50], we sacrificed the animal using an intracoelomic injection of sodium pentobarbitol (100 mg/kg). We then removed the venom glands and stored them separately in either RNAlater (Thermo Fisher Scientific, Waltham, MA, USA) at 4 ${}^{\circ }$C overnight or in liquid nitrogen before moving to −80 ${}^{\circ }$C for long-term storage. Each specimen was fixed in 10% buffered formalin for five days and then transfered to 70% ethanol and deposited in a natural history museum (Table 1).

### 5.3. Venom Type Determination

We determined the venom type of each individual using reverse-phased high performance liquid chromatography (RP-HPLC) similar to Margres et al. [51]. We resuspended the dry venom in water and removed insoluble material via centrifugation. We determined the concentration of the venom utilizing the Qubit Protein Assay (Thermo Fisher Scientific) following the manufacturer’s protocol on a Qubit 3.0 Fluorometer (Thermo Fisher Scientific). We then injected 100 μg of venom onto a Jupiter C18 column (250 × 2 mm; Phenomenex, Torrence, CA, USA) using two solvents: A = 0.1% trifluoroacetic acid (TFA) in water and B = 0.075% TFA in acetonitrile. RP-HPLC was conducted on a Beckman System Gold HPLC (Beckman Coulter, Fullerton, CA, USA). The gradient began with 95% A and 5% B for 5 min followed by a 1% per minute linear gradient to 25% B. This was followed by a 0.25% per minute linear gradient to 55% B, a 2% per minute linear gradient to 75% B, a 14% per minute linear gradient to 5% B, and then 5 min at the initial conditions all at a 0.2 mL/min flow rate. Total run time was 180 min for each sample and the effluent was monitored at 220 and 280 nm [52].

We were able to distinguish Type A and Type B venoms from each other based on the presence or absence of MTX and SVMPs based on previous RP-HPLC profiles in *C. scutulatus* [39] and under these conditions [28]. We selected a total of nine individuals, five with Type A venom and four with Type B venom (Figure 2), for venom gland transcriptome sequencing. The Type A individuals were selected to maximize the geographic breadth around the Type B zone in central Arizona (Figure 1).

### 5.4. Venom Gland Transcriptome Sequencing

For each transcriptome animal, total RNA was extracted from the left and right venom glands independently using a TRIzol-based RNA extraction as previously described [52]. We first diced approximately 100 mg of venom gland tissue into small pieces and added it to 500 μL of TRIzol (Invitrogen, Carlsbad, CA, USA). We aspirated the tissue and TRIzol through a 20-gauge needle at least 10× until the tissue was homogenized. We added 500 μL of fresh TRIzol and added the entire solution to Phase Lock Gel Heavy tubes (5Prime 2302830, Quantabio, Beverly, MA, USA). We added 200 μL of 20% chloroform, shook the solution, and centrifuged for 20 min after three minutes of incubation to separate the RNA. RNA was isolated with isopropyl alcohol precipitation overnight at −20 ${}^{\circ }$C and washed with 75% ethanol. We estimated the concentration of total RNA using the Qubit RNA BR Assay (Thermo Fisher Scientific) and evaluated the quality and final concentration using an Agilent Bioanalyzer 2100 with an RNA 6000 Pico Kit (Agilent Technologies, Santa Clara, CA, USA) following the manufacturer’s instructions. We then combined RNA from both glands in equal concentration for library preparation as there is no difference in the transcriptome between the two glands [53].

We used 1 μg of total RNA for mRNA isolation and cDNA library preparation [54,55]. We used the New England Biolabs (NEB, Ipswich, MA, USA) NEBNext Poly(A) mRNA magnetic isolation kit (E7490S), the NEBNext Ultra RNA Library Prep Kit for Illumina (E7530), and the NEBNext Multiplex Oligos for Illumina (E7335 -Index primer set 1) following the manufacturer’s protocols with a target mean insert size of 370 bp, a fragmentation time of 15.5 min, and 14 PCR cycles in the final enrichment step to yield the appropriate cDNA concentration. To do this, we isolated RNA with poly-A tails from the total RNA with oligo-dT beads and then immediately moved to first and second strand cDNA synthesis [56]. We used Agencourt AMPure XP PCR Purification Beads (Beckman Coulter) to purify DNA throughout the protocol. We estimated the concentration of our DNA library using the Qubit DNA BR Assay (Thermo Fisher Scientific) and evaluated the quality and final concentration using the Bioanalyzer with an HS DNA Kit following the manufacturer’s instructions (Agilent Technologies). KAPA qPCR was conducted at the Florida State University Molecular Cloning Facility to determine the amplifiable concentration of each sample. For the samples sequenced on the Illumina MiSeq in the Florida State University Department of Biological Science DNA Sequencing Facility, we used the MiSeq Version 2 Reagent Kit and sequenced the individuals separately except for CLP 2136 and CLP 2142 which were pooled using equal amounts of amplifiable cDNA for each. If the sample was sequenced on the Illumina HiSeq 2500 in the Florida State University College of Medicine Translational Science Laboratory, we used the KAPA result to pool the samples to a final concentration of 10 nM so that each library was equally represented. We assessed the concentration and quality of the pooled DNA sample on the Bioanalyzer with the HS DNA Kit and performed an additional round of KAPA PCR before sequencing. Both the MiSeq and HiSeq runs were 150 bp paired-end reads (Table 1) [28].

### 5.5. Transcriptome Assembly

We first used the Illumina quality filter to remove low-quality reads, and because the insert sizes for cDNA libraries were approximately 370 bp (170 bp without adapters), we were able to merge the remaining overlapping 150-nt paired-end reads at their 3’ ends with PEAR v 0.9.6 [57]. Merged reads were then used for subsequent assemblies and analyses. We assembled venom gland transcriptomes as described in Rokyta et al. [53]. To maximize the number of transcripts recovered, we assembled the merged reads using DNAStar SeqMan NGen version 12.3.1 (DNASTAR Inc., Madison, WI, USA) with default settings except that only contigs with ≥200 assembled reads were kept and Extender [49]. We then compared all assembled transcripts through both methods to a venom toxin and nontoxin sequence database based on transcripts identified previously [27,28,49,52,53]. This database included 1047 toxins and 4516 nontoxins. Toxins were annotated if they matched at ≥90% to the local database based on similarity in Cd-hit-est v.4.6 [58] and had at least 10× coverage across the transcript. The remaining unmatched contigs were compared via blastx v. 2.2.30+ searches (minimum e-value of 10${}^{-4}$) to the curated Uniprot animal toxins database (downloaded 16 November 2017) and annotated in Geneious v 10.1.2 (Biomatters Ltd., Auckland, New Zealand) by checking for open reading frames that matched the blastx search [8,27,28,49]. Signal peptides were checked for and identified with SignalP v 4.1 [59,60].

The final set of possible toxin and nontoxin transcripts for each of the nine individuals included anything that matched our local database or the Uniprot database that also had a complete protein coding sequences. From these, we extracted the coding sequence from each transcript in Geneious and then removed duplicates using the BBtools package Dedupe (Joint Genome Institute, Department of Energy, Walnut Creek, CA, USA) implemented in Geneious. We screened for and removed chimeric sequences in our toxins within each individual in two ways. First, we mapped the merged reads to the identified transcripts using Bowtie2 v 2.3.0 [42] implemented in Geneious and identified ones with irregular coverage including zero, multimodal, or uneven coverage. Second, we checked for recombination within each of the toxin families through the ClustalW alignment algorithm [61] implemented in Geneious and identified transcripts that matched exactly with two or more other sequences. Transcripts identified in both were removed. The toxin and nontoxin transcripts remaining were clustered using Cd-hit at ≥98% (-c .98) and a representative transcript was retained for each cluster. We then combined all toxin and nontoxins from the nine individuals into two sets based on their venom type. We removed duplicates, chimeras, and clustered as described above to get a master transcriptome for both Type A and Type B *C. scutulatus*. Finally, these were combined, had duplicates and chimeras removed, and clustered to get a master *C. scutulatus* transcriptome for final analyses.

### 5.6. Expression Analysis

Relative expression of toxin and nontoxin genes was calculated by mapping merged reads to the final transcript set with Bowtie2 [42,62] in RSEM v 1.3.0 [41] on the Stokes HPC on the UCF Advanced Research Computing Center using the default parameters. We used the transcripts per million reads (TPM) data for each individual as our abundance estimates [11]. We imported the dataset into RStudio v. 1.1.383 using R v. 3.4.2 (R Development Core Team 2006). We then created a 10th and 11th “individual” by averaging the TPM values for the five A animals (Average A) and four B animals (Average B). To eliminate 0 values from the dataset while preserving the compositional nature of expression data, we used the cmultRepl function of the R package zCompositions [63] then performed a centered log-ratio (clr) transformation to linearize the compositional dataset while preserving rank order of the transcripts [11,64].

To determine if there was differential expression of toxin genes within and among venom types, we performed pairwise comparisons of each of the nine individuals as well as the Average A and Average B transcriptomes. We used the nontoxin expression values to generate a null distribution of expression divergence [53]. This was done by taking the absolute value of the difference in the transformed data for the two individuals being compared and finding the 99th percentile value. Any toxin outside of this value was identified as an outlier to the null distribution. For each pairwise comparison, we used the Spearman’s rank correlation coefficient (ρ), Pearson’s correlation coefficient (R), and a coefficient of determination (R${}^{2}$) to look at how similar the individuals being compared were. Finally, we tested for differential expression between our two venom types and between males and females using DESeq v 1.26.0 [65] and DESeq2 v. 1.14.1 [66] with a false discovery rate threshold of 0.1. We used the expected counts generated in RSEM and used the effective length to normalize the data. Toxins were assigned names based on toxin family and then ranked in order of highest average expression across all individuals for all families except PLA${}_{2}$s. These were named to match the PLA${}_{2}$s identified by Dowell et al. [7].

In RSEM, it is possible for a transcript that is not in the transcriptome to have a non-zero value. This is due to unmapped reads mapping to dissimilar sequences or, more commonly, highly similar regions among toxins within a specific family having reads dispersed among the different representatives. To determine which toxin transcripts were present in each individual and not an artifact of poor mapping, we aligned merged reads for each individual to the *C. scutulatus* master transcriptome using Bowtie2 implemented in Geneious. Any toxin that had more than 10% of the sequence with less than 5× coverage were considered absent in the transcriptome for that individual.

### 5.7. PLA${}_{2}$ Diversity

We tested the Dowell et al. [7] hypothesis of PLA${}_{2}$ gene loss in *C. scutulatus* by assessing the diversity of PLA${}_{2}$s in the nine individuals. We downloaded the four published genome fragments from GenBank (KX211993-KX21996) and extracted the mRNA sequence from all annotated PLA${}_{2}$s. We clustered all sequences using Cd-hit at ≥90% (-c .90) and a representative transcript was retained for each cluster. We then used Bowtie2 to align merged reads from each individual to the 10 resulting PLA${}_{2}$s to determine which of the PLA${}_{2}$s described in Dowell et al. [7] were present in *C. scutulatus*. We followed the same rule as above where there could not be less than 5× coverage over 10% of the transcript.

### 5.8. Data Availability

Raw data for the nine venom gland transcriptomes were submitted to the National Center for Biotechnology Information (NCBI) Sequence Read Archive (SRA) accession SRP011323. BioSample accession numbers are provided in Table 1 and are under BioProject PRJNA88989. The consensus transcriptome was submitted to the NCBI Transcriptome Shotgun Assembly (TSA) database. This TSA project has been deposited at DDBJ/EMBL/GenBank under the accession GGIP00000000. The version described in this paper is the first version, GGIP01000000.

## Figures and Tables

**Table 1:** Sample information for the nine specimens of *C. scutulatus* sequenced from the U.S. Sonoran Desert. ASNHC = Angelo State Natural History Collection, San Angelo, Texas; ASU = Arizona State University Natural History Museum, Tempe, Arizona; CLP = Christopher L. Parkinson field number.

Specimen	Museum	Venom	Sex	SVL	Mass	State	County	Sequencing	Read	Merged	BioSample ${}^{1}$
ID	ID	Type		(mm)	(g)			Platform	Pairs	Reads	Accession
CLP1930	ASNHC14997	A	F	724	195	NM	Hidalgo	MiSeq	15,649,085	13,083,925	SAMN08596271
CLP1936	ASU36035	A	F	441	48	AZ	Graham	MiSeq	20,835,668	18,182,033	SAMN08596272
CLP1959	ASU36061	A	M	730	204	AZ	Yavapai	MiSeq	20,577,779	17,091,930	SAMN08596273
CLP1961	ASU36062	A	M	564	90	AZ	Yavapai	HiSeq	11,929,639	10,061,262	SAMN08596274
CLP1972	ASU36092	A	M	635	126	AZ	Pima	MiSeq	13,168,704	11,521,499	SAMN08596275
CLP1831	ASU36089	B	F	795	344	AZ	Pima	HiSeq	15,448,552	13,526,047	SAMN08596267
CLP1835	ASU36102	B	F	685	146	AZ	Pinal	HiSeq	16,271,477	14,210,557	SAMN08596269
CLP2136	ASU36103	B	M	1030	627	AZ	Pinal	MiSeq	7,771,613	6,864,270	SAMN08596277
CLP2142	ASU36104	B	M	775	262	AZ	Pinal	MiSeq	10,039,268	8,893,097	SAMN08596278

${}^{1}$ National Center for Biotechnology Information under BioProject PRJNA88989.

**Table 2:** Transcripts per million reads (TPM) values for 75 toxins identified in the nine *C. scutulatus* individuals. TPM values were generated in RSEM [41] with Bowtie2 [42]. CRISP: cysteine-rich secretory protein; BPP: bradykinin potentiating peptide; CTL: C-type lectin; HYAL: hyaluronidase; KUN: Kunitz peptide; LAAO: L-amino-acid oxidase; MYO: myotoxin; NGF: nerve growth factor; NUC: 5’ nucleotidase; PDE: phosphodiesterase; PLB: phospholipase B; SVMP: snake venom metalloproteinase; SVSP: snake venom serine protease; VEGF: vascular endothelial growth factor.

Toxin	Type A					Type B			
	CLP1930	CLP1936	CLP1959	CLP1961	CLP1972	CLP1831	CLP1835	CLP2136	CLP2142
BPP-1	140,915.97	42,576.13	83,149.6	63,491.95	45,783.43	152,393.13	126,252.15	206,001.59	140,706.64
CRISP-1	4835.29	4825.49	15,386.6	7108.63	5075.55	4047.71	4625.53	2879.87	3386.38
CTL-1	0.00	3.65	14,629.20	25,897.00	0.00	17,494.71	26,507.89	18,545.42	44,489.80
CTL-2	0.00	1.98	12,439.65	20,441.04	0.00	18,825.37	22,039.29	18,679.67	43,789.90
CTL-3	1.12	0.00	0.31	10.11	0.00	12,805.18	13,046.93	13,965.96	21,899.71
CTL-4	0.00	0.45	0.00	3.20	0.00	12,605.98	15,574.61	11,584.92	16,750.81
CTL-5	0.41	2.52	7965.07	10,951.63	0.00	4218.11	11,546.02	10,057.03	10,667.89
CTL-6	0.40	1.26	11,399.06	12,437.39	0.00	3461.60	3578.61	9394.12	10,374.16
CTL-7	0.38	1.44	7423.12	8508.27	0.00	4653.94	10,065.04	9327.24	10,662.63
CTL-8	22.39	8.78	7498.38	9084.97	232.76	2273.74	4494.79	6799.15	11,587.90
CTL-9	3859.43	4539.36	9932.82	10,002.06	3012.54	1561.25	2.48	1.52	2634.56
CTL-10	10.63	4.93	7658.06	9102.48	84.69	2499.90	3293.65	6962.90	3823.74
CTL-11	0.00	0.47	2744.48	3752.74	0.00	2814.96	6914.87	4523.03	5131.21
CTL-12	3604.05	3074.72	6797.37	6941.60	2123.74	1975.31	14.78	24.72	40.48
CTL-13	0.00	0.41	2379.11	2630.23	0.00	947.76	3008.94	1948.93	2122.28
CTL-14	0.88	171.20	883.68	555.05	147.92	637.66	1994.47	2157.31	2572.39
CTL-15	995.02	292.59	809.42	726.11	910.53	1168.87	748.84	1791.77	1309.00
CTL-16	1246.38	418.83	2132.33	2473.36	472.09	433.29	0.55	234.84	342.16
CTL-17	0.00	0.00	0.41	8.06	0.00	4.34	10.41	51.78	6202.50
CTL-18	325.45	305.49	4026.88	247.89	201.87	279.93	274.45	36.75	527.21
CTL-19	28.46	1045.78	2968.50	60.46	395.23	49.87	213.36	285.33	172.05
CTL-20	0.00	0.00	0.00	0.87	0.00	0.00	0.00	1.60	1233.72
CTL-21	0.40	0.00	0.53	11.61	0.00	59.06	27.62	511.53	361.34
CTL-22	0.00	0.00	0.38	29.57	0.00	51.73	54.86	280.15	438.68
CTL-23	3.52	9.49	15.25	7.90	45.56	5.09	13.42	1.34	9.87
HYAL-1	557.54	304.13	1309.25	379.62	221.07	468.14	408.71	436.41	222.96
KUN-1	178.35	48.25	168.92	123.70	71.34	107.63	147.09	195.49	122.11
KUN-2	14.96	4.32	13.36	14.48	5.19	20.25	20.77	20.98	12.38
LAAO-1	5188.99	2817.21	20,588.57	9120.95	1626.36	5792.61	10,578.38	6868.00	5503.11
MYO-1	1220.73	549,957.26	1072.89	27.84	457,306.12	209,616.39	44.93	44.14	29,489.77
MYO-2	6119.03	49,381.65	2467.88	3325.84	95,421.49	27,536.10	2903.51	6987.97	3925.07
MYO-3	0.00	0.00	0.00	34,019.55	0.00	0.00	87,549.28	30.87	33,694.44
MYO-4	5843.47	3702.71	12,274.75	19,693.00	497.74	1611.10	10,899.95	2894.17	4002.29
MYO-5	0.00	82.14	0.00	0.00	0.00	44.63	0.00	43.19	35,485.73
MYO-6	0.00	0.00	0.00	0.00	0.00	0.00	0.00	0.00	2706.33
MYO-7	0.00	0.00	0.00	0.00	0.00	0.00	0.00	0.00	1805.60
MYO-8	3.83	960.47	8.14	0.00	0.00	0.00	0.00	0.00	0.00
NGF-1	2021.69	761.53	5039.75	2527.51	2023.71	1592.10	2240.15	2459.36	2108.54
NUC-1	779.38	366.04	1526.31	945.96	368.05	610.32	960.93	1068.46	502.67
PDE-1	445.18	229.67	691.74	312.13	236.13	209.66	277.73	208.12	249.61
Pla2gA1	25,306.99	39,764.45	61.41	46.36	2.19	4.48	50,036.23	0.00	0.61
Pla2gB1	3.44	7.16	29,405.58	14.34	1.43	37,548.76	46,634.43	51,949.04	31,992.11
Pla2gK	0.00	0.30	1793.61	3.04	0.00	1356.66	2971.25	1123.48	807.59
Pla2gA2-MTXA	274,390.23	80,733.96	102,588.14	199,107.20	102,677.33	4.93	65.65	0.00	0.00
Pla2gB2-MTXB	133,822.75	43,410.96	48,028.34	138,470.84	42,571.19	3.73	30.28	0.00	0.00
PLA${}_{2}$-6	21,306.42	22.86	106,639.29	90,911.79	51,893.58	60,760.91	56,428.98	129,974.00	93,064.76
PLB-1	837.02	189.30	1170.66	1059.23	597.70	509.37	745.22	346.61	182.83
SVMPII-1	0.00	0.84	0.60	122.86	3.30	23,801.48	32,375.22	24,762.53	24,369.30
SVMPIII-1	0.00	1.20	1.83	262.28	9.58	58,107.40	69,839.79	69,573.48	39,410.80
SVMPIII-2	8.60	3074.27	61.30	2524.10	479.43	3581.65	14,465.14	2364.36	4089.81
SVMPIII-3	0.01	0.79	1.32	206.34	6.17	6920.71	8968.12	7129.26	6456.84
SVMPIII-4	1497.61	1018.71	4893.89	2711.96	276.26	3637.08	4729.55	5060.59	2488.36
SVMPIII-5	6.65	4.39	4.51	2.02	0.00	1293.10	2689.88	3646.35	1942.60
SVMPIII-6	19.52	12.50	6.89	9.20	2.20	274.19	3250.08	2739.21	663.84
SVMPIII-7	0.02	0.17	0.57	16.36	0.53	643.82	2557.44	2534.21	994.18
SVMPIII-8	0.00	0.00	0.00	0.07	0.00	44.33	50.02	4977.26	25.70
SVMPIII-9	0.00	0.00	0.00	0.07	0.00	279.91	2000.74	1017.78	1092.48
SVMPIII-10	0.00	0.01	0.00	0.19	0.00	181.26	751.33	220.24	404.67
SVSP-1	25,158.51	15,910.07	30,344.55	17,038.01	10,432.56	21,732.91	32,652.46	20,711.71	26,686.11
SVSP-2	42,979.80	20,190.56	35,325.26	21,389.97	14,037.74	16,208.71	10,861.87	6624.20	6521.70
SVSP-3	24,402.09	7206.88	34,812.15	9121.20	3774.14	19,942.19	20,070.68	20,385.25	15,388.82
SVSP-4	29,001.28	5094.40	18,056.16	9305.84	1916.43	8654.88	9431.62	7042.42	8412.05
SVSP-5	37,090.47	5414.68	21,446.17	8874.54	1346.38	1863.68	5619.86	2373.32	3563.03
SVSP-6	11,830.88	7170.10	15,765.87	9603.42	4485.31	8500.16	12,274.97	6642.73	10,481.74
SVSP-7	7289.06	2382.92	5895.10	3081.90	1513.33	4024.78	2685.70	1659.02	1379.27
SVSP-8	4813.04	2087.12	5900.27	2021.75	407.33	2169.99	2985.67	1994.54	1945.35
SVSP-9	4048.20	724.15	1391.55	1288.30	1231.13	3097.60	3252.51	2154.34	3169.89
SVSP-10	4038.45	1189.72	4293.59	1766.03	84.61	1208.22	2752.40	2430.80	2459.42
SVSP-11	5777.06	1464.77	2503.34	1599.56	962.33	2311.84	3050.76	478.40	1664.01
SVSP-12	3674.93	1456.06	3014.74	2310.02	565.32	2209.80	1897.49	1761.75	1281.51
SVSP-13	3238.56	798.05	3159.21	1118.98	287.65	1467.22	709.22	982.38	1198.93
SVSP-14	1558.37	620.40	1082.12	667.47	38.63	762.05	289.19	599.21	693.61
VEGF-1	4255.45	10,557.63	14,271.01	11,732.75	7493.65	20,763.31	9620.66	12,596.93	10,796.52
VEGF-2	134.91	18.84	82.27	95.20	3.90	96.12	72.87	313.81	91.89
Vespryn-1	4976.55	718.69	3827.06	1024.92	1089.92	2106.99	2068.47	922.31	582.28

**Table 3:** Presence and absence data for toxin transcripts that were not found in all individuals. Toxins highlighted in dark blue were found in all Type B individuals but never in Type A individuals. Toxins highlighted in dark red were found in all Type A individuals but never in Type B individuals. Toxins highlighted in light blue or light red were only found in individuals of that venom type but were not found in all individuals. The last row is the number (out of 75) of toxins present in total, which includes 33 toxins present in all individuals not listed in this table. To be present in the transcriptome, toxins had to have at least 5× coverage over 90% of the transcript. MTXA: acidic subunit of Mojave toxin; MTXB: basic subunit of Mojave toxin.

Toxin	Type A					Type B			
	CLP1930	CLP1936	CLP1959	CLP1961	CLP1972	CLP1831	CLP1835	CLP2136	CLP2142
CTL-1	-	-	+	+	-	+	+	+	+
CTL-2	-	-	+	+	-	+	+	+	+
CTL-3	-	-	-	-	-	+	+	+	+
CTL-4	-	-	-	-	-	+	+	+	+
CTL-5	-	-	+	+	-	+	+	+	+
CTL-6	-	-	+	+	-	+	+	+	+
CTL-7	-	-	+	+	-	+	+	+	+
CTL-8	-	-	+	+	+	+	+	+	+
CTL-9	+	+	+	+	+	+	-	-	+
CTL-10	-	-	+	+	+	+	+	+	+
CTL-11	-	-	+	+	-	+	+	+	+
CTL-12	+	+	+	+	+	+	+	-	-
CTL-13	-	-	+	+	-	+	+	+	+
CTL-14	-	+	+	+	+	+	+	+	+
CTL-16	+	+	+	+	+	+	-	+	+
CTL-17	-	-	-	-	-	-	-	+	+
CTL-19	+	+	+	+	+	-	+	-	+
CTL-20	-	-	-	-	-	-	-	-	+
CTL-21	-	-	-	-	-	+	+	+	+
CTL-22	-	-	-	-	-	+	+	+	+
CTL-23	+	+	+	+	+	+	+	-	+
MYO-3	-	-	-	+	-	-	+	+	+
MYO-5	-	+	-	-	-	+	-	+	+
MYO-6	-	-	-	-	-	-	-	-	+
MYO-7	-	-	-	-	-	-	-	-	+
MYO-8	-	+	+	-	-	-	-	-	-
Pla2gA1	+	+	+	-	-	-	+	-	-
Pla2gB1	-	-	+	-	-	+	+	+	+
Pla2gK	-	-	+	-	-	+	+	+	+
Pla2gA2-MTXA	+	+	+	+	+	-	-	-	-
Pla2gB2-MTXB	+	+	+	+	+	-	-	-	-
PLA${}_{2}$-6	+	-	+	+	+	+	+	+	+
SVMPII-1	-	-	-	-	-	+	+	+	+
SVMPIII-1	-	-	-	-	-	+	+	+	+
SVMPIII-2	-	+	+	+	+	+	+	+	+
SVMPIII-3	-	-	-	-	-	+	+	+	+
SVMPIII-5	-	-	-	-	-	+	+	+	+
SVMPIII-6	-	-	-	-	-	+	+	+	+
SVMPIII-7	-	-	-	-	-	+	+	+	+
SVMPIII-8	-	-	-	-	-	-	-	+	-
SVMPIII-9	-	-	-	-	-	+	+	+	+
SVMPIII-10	-	-	-	-	-	+	+	+	+
Toxins Present	42	45	56	53	45	64	66	64	69

**Table 4:** Differential expression analyses for toxins between the nine Type A and Type B *C. scutulatus* as well as the average Type A (AveA) and Type B (AveB) transcriptomes. The UpB and UpA count data were generated by identifying outlier transcripts in the pairwise comparisons of the Type A and Type B individuals (maximum of 20 comparisons). The last four columns are the data from DESeq and DESeq2 identifying differential expression between the two venom types. Toxins highlighted in dark red were found in all Type A individuals but never in Type B individuals. Toxins highlighted in light blue or light red were exclusively found in individuals of that venom type but were not found in all individuals. Toxins highlighted in green were found in all nine individuals. Toxins with NA for *P*${}_{\mathrm{adj}}$ only had one individual in one of the treatments with the toxin so it was not possible to calculate in DESeq2.

Toxin	UpB	UpA	Δ ${}_{B-A}$	B to A	AveB to AveA	Log${}_{2}$Δ DESeq1	*P*${}_{\mathrm{adj}}$ DESeq1	Log${}_{2}$Δ DESeq2	*P*${}_{\mathrm{adj}}$ DESeq2
CTL-3	20	0	20	Up	Up	12.76	6.55 × 10${}^{-42}$	12.73	1.11 × 10${}^{-20}$
CTL-4	20	0	20	Up	Up	14.14	5.91 × 10${}^{-96}$	14.13	1.03 × 10${}^{-111}$
SVMPII-1	20	0	20	Up	Up	9.94	1.96 × 10${}^{-71}$	9.97	N/A
SVMPIII-1	20	0	20	Up	Up	9.97	1.01 × 10${}^{-71}$	10.00	N/A
SVMPIII-10	20	0	20	Up	Up	13.17	6.48 × 10${}^{-16}$	13.28	4.27 × 10${}^{-40}$
SVMPIII-3	20	0	20	Up	Up	7.35	2.89 × 10${}^{-48}$	7.38	N/A
SVMPIII-5	20	0	20	Up	Up	8.96	8.76 × 10${}^{-42}$	8.96	4.42 × 10${}^{-16}$
SVMPIII-6	20	0	20	Up	Up	6.86	5.44 × 10${}^{-04}$	6.86	5.30 × 10${}^{-22}$
SVMPIII-7	20	0	20	Up	Up	8.75	6.91 × 10${}^{-16}$	8.79	1.00 × 10${}^{-08}$
SVMPIII-9	20	0	20	Up	Up	16.23	1.76 × 10${}^{-20}$	15.89	2.41 × 10${}^{-48}$
CTL-21	19	0	19	Up	Up	6.65	2.60 × 10${}^{-02}$	6.58	3.92 × 10${}^{-03}$
Pla2gB1	19	0	19	Up	Up	3.06	6.07 × 10${}^{-08}$	3.04	N/A
CTL-22	18	0	18	Up	Up	-	-	-	-
CTL-14	16	0	16	Up	Up	2.22	9.75 × 10${}^{-02}$	-	-
Pla2gK	16	0	16	Up	Up	-	-	2.32	N/A
CTL-1	15	0	15	Up	Up	-	-	-	-
CTL-2	15	0	15	Up	Up	-	-	-	-
CTL-11	14	0	14	Up	Up	2.01	7.05 × 10${}^{-02}$	-	-
SVMPIII-2	14	0	14	Up	Up	-	-	-	-
CTL-7	12	0	12	Up	Up	-	-	-	-
CTL-17	10	0	10	Up	Up	-	-	10.07	N/A
MYO-5	9	1	8	Up	Up	-	-	8.04	N/A
MYO-3	9	2	7	Up	Up	-	-	-	-
CTL-20	5	0	5	Up	Up	-	-	11.01	N/A
MYO-6	5	0	5	Up	Up	-	-	23.74	N/A
MYO-7	5	0	5	Up	Up	-	-	23.49	N/A
SVMPIII-8	5	0	5	Up	Up	-	-	15.96	N/A
MYO-2	4	3	1	Up	Down	-	-	-	-
CRISP-1	0	0	0	No Difference	-	-	-	−1.32	3.16 × 10${}^{-03}$
CTL-19	6	8	−2	Down	Down	-	-	-	-
MYO-1	7	9	−2	Down	Down	-	-	-	-
PLB-1	0	2	−2	Down	-	-	-	−1.07	7.76 × 10${}^{-02}$
SVSP-2	1	4	−3	Down	Down	−1.77	9.50 × 10${}^{-03}$	−1.77	5.11 × 10${}^{-04}$
MYO-8	0	4	−4	Down	Down	-	-	−23.22	N/A
SVSP-5	1	5	−4	Down	Down	-	-	−2.32	5.29 × 10${}^{-03}$
CTL-18	0	5	−5	Down	Down	-	-	-	-
CTL-23	0	7	−7	Down	Down	-	-	-	-
CTL-16	0	9	−9	Down	Down	−2.49	5.78 × 10${}^{-04}$	-	-
CTL-9	0	11	−11	Down	Down	−2.73	2.56 × 10${}^{-06}$	-	-
CTL-12	0	15	−15	Down	Down	−3.36	1.14 × 10${}^{-09}$	−3.35	7.88 × 10${}^{-02}$
Pla2gA2-MTXA	0	20	−20	Down	Down	−13.55	8.76 × 10${}^{-42}$	−13.51	1.18 × 10${}^{-35}$
Pla2gB2-MTXB	0	20	−20	Down	Down	−13.65	5.75 × 10${}^{-38}$	−13.61	2.10 × 10${}^{-34}$

**Table 5:** Presence or absence data for PLA${}_{2}$s identified by Dowell et al. [7] (the first five PLA${}_{2}$s) and the sixth PLA${}_{2}$ identified in this study. The first four individuals were the specimen used in Dowell et al. [7] and only presence/absence is indicated. The last nine individuals are *C. scutulatus* specimens sequenced in this study with venom type indicated and transcripts per million reads (TPM) values given when that PLA${}_{2}$ is present. The one TPM value denoted by an * had eight nonsynonymous nucleotide changes in the sequence compared to the other three *C. scutulatus* specimens and matched that of *Crotalus viridis* (Genbank accession AF403134).

Specimen	Pla2gA1	Pla2gB1	Pla2gK	Pla2gA2-MTXA	Pla2gB2-MTXB	PLA${}_{2}$-6
*C. atrox*	+	+	+	-	-	-
*C. atrox*	+	+	+	-	-	-
*C. adamanteus*	+	+	-	-	-	-
*C. scutulatus*	+	-	-	+	+	-
CLP1930A	55,212.77	-	-	603,261.66	294,142.52	47,256.79
CLP1936A	242,536.57	-	-	492,462.39	264,770.94	-
CLP1959A	214.27	101,885.11	6162.67	355,273.29	166,449.59	369,588.5
CLP1961A	-	-	-	464,139.64	323,205.86	212,285.6
CLP1972A	-	-	-	520,518.57	216,000.5	263,309.5
CLP1831B	-	370,853.15	13,305.52	-	-	600,310.86
CLP1835B	320,034.14 *	297,244.14	18,761.21	-	-	58,732.73
CLP2136B	-	283,162.92	6014.01	-	-	709,003.82
CLP2142B	-	253,634.76	6285.41	-	-	738,317.63

## Abbreviations

The following abbreviations are used in this manuscript:

ARCC	Advanced Research Computing Center
BPP	Bradykinin potentiating peptide
clr	centered log-ratio
CLP	Christopher L. Parkinson field number
CRISP	Cysteine-rich secretory protein
CTL	C-type lectin
FSU	Florida State University
HYAL	Hyaluronidase
HPC	High Performance Cluster
KUN	Kunitz peptide
LAAO	L-amino-acid oxidase
LD${}_{50}$	Lethal dose${}_{50}$
MDPI	Multidisciplinary Digital Publishing Institute
MTX	Mojave toxin
MTXA	Acidic (α) subunit of Mojave toxin
MTXB	Basic (β) subunit of Mojave toxin
MYO	Myotoxin-*a*
NAHR	Non-allelic homologous recombination
NGF	Nerve growth factor
NUC	5${}^{\prime }$ nucleotidase
PDE	Phosphodiesterase
PLA${}_{2}$	Phospholipase A${}_{2}$
PLB	Phospholipase B
RP-HPLC	Reverse-phased high performance liquid chromatography
SVMP	Snake venom metalloproteinase
SVSP	Snake venom serine protease
TPM	Transcripts per million reads
VEGF	Vascular endothelial growth factor
UCF	University of Central Florida
